# Increased circulating leukocyte numbers and altered macrophage phenotype correlate with the altered immune response to brain injury in metallothionein (MT) -I/II null mutant mice

**DOI:** 10.1186/1742-2094-8-172

**Published:** 2011-12-07

**Authors:** Michael W Pankhurst, William Bennett, Matthew TK Kirkcaldie, Adrian K West, Roger S Chung

**Affiliations:** 1 Menzies Research Institute Tasmania, University of Tasmania, 17 Liverpool Street, Hobart, Tasmania, Australia; 2 Department of Anatomy, University of Otago, 270 Great King St, Dunedin, New Zealand; 3 School of Medicine, University of Tasmania, 17 Liverpool Street, Hobart, Tasmania, Australia

**Keywords:** Metallothionein, cryolesion, brain injury, alternatively activated macrophages

## Abstract

**Background:**

Metallothionein-I and -II (MT-I/II) is produced by reactive astrocytes in the injured brain and has been shown to have neuroprotective effects. The neuroprotective effects of MT-I/II can be replicated *in vitro *which suggests that MT-I/II may act directly on injured neurons. However, MT-I/II is also known to modulate the immune system and inflammatory processes mediated by the immune system can exacerbate brain injury. The present study tests the hypothesis that MT-I/II may have an indirect neuroprotective action via modulation of the immune system.

**Methods:**

Wild type and MT-I/II^-/- ^mice were administered cryolesion brain injury and the progression of brain injury was compared by immunohistochemistry and quantitative reverse-transcriptase PCR. The levels of circulating leukocytes in the two strains were compared by flow cytometry and plasma cytokines were assayed by immunoassay.

**Results:**

Comparison of MT-I/II^-/- ^mice with wild type controls following cryolesion brain injury revealed that the MT-I/II^-/- ^mice only showed increased rates of neuron death after 7 days post-injury (DPI). This coincided with increases in numbers of T cells in the injury site, increased IL-2 levels in plasma and increased circulating leukocyte numbers in MT-I/II^-/- ^mice which were only significant at 7 DPI relative to wild type mice. Examination of mRNA for the marker of alternatively activated macrophages, Ym1, revealed a decreased expression level in circulating monocytes and brain of MT-I/II^-/- ^mice that was independent of brain injury.

**Conclusions:**

These results contribute to the evidence that MT-I/II^-/- ^mice have altered immune system function and provide a new hypothesis that this alteration is partly responsible for the differences observed in MT-I/II^-/- ^mice after brain injury relative to wild type mice.

## Background

Metallothionein (MT) is a 6-7 kDa, cysteine-rich, zinc-binding protein that has antioxidant properties. MT-I and MT-II are similar isoforms, often considered to behave as one species (MT-I/II), that share the ability to provide protection to the injured brain. During insult to the central nervous system (CNS), metallothionein-I and -II double knockout (MT-I/II^-/-^) mice show increased neuron death or larger injury size after brain injury [1-3]. This firmly suggests that the presence of MT-I/II provides protection against CNS perturbation but the precise mechanisms that underlie this are yet to be identified. *In vitro *experiments have demonstrated that MT-I/II can provide protection, directly to neurons, against zinc toxicity [4] and can protect astrocytes from oxidative stress [5]. In a regenerative context, MT-I/II can enhance neurite extension in neurons [6]. However, a defining characteristic of brain injury in MT-I/II^-/- ^mice is the increased numbers of inflammatory cells such as microglia or macrophages, and T cells compared to wild type mice [2,7,8]. Notably, MT-I/II has been shown to affect immune system processes such as immunoglobulin production [9-14]. Leukocytes infiltrate the injured CNS and have the potential to be neurotoxic which makes it difficult to determine if the increased cell death observed in the injured brains of MT-I/II^-/- ^mice is due to the absence of the neuroprotective effects of MT-I/II in the CNS or the absence of the modulatory effects that MT-I/II has on the immune system.

The infiltration of neutrophils into CNS injuries is the most rapid of any type of leukocyte but neutrophils do not persist beyond 2 days post-injury, at which time monocytes become the dominant infiltrating leukocyte [15]. T cell infiltration occurs in several waves with an early infiltration within 1 hour [16], followed by a second infiltration at 24 hours [17]. However, the maximal T cell occupation of the injured CNS begins to occur about 1 week after the initial injury [18]. Evidence suggests that many immune system processes, such as inflammatory cytokine production and the oxidative burst, are neurotoxic and can impede the resolution of brain injury [19-21]. It is feasible that the number of immune cells entering the CNS can influence the progression of brain injury but the phenotype of the immune cells may also affect this process. For example, naïve helper T cells can take on one of several phenotypes when they first become activated; the predominant types are type 1 helper T cell phenotype and the type 2 helper T cell phenotype [22]. Th1 cells promote the formation of classically activated macrophages (caMΦs) and augment the production of pro-inflammatory cytokines and reactive oxygen species and other neurotoxic molecules whereas Th2 cells promote formation of alternatively activated macrophages (aaMΦs) which antagonise these processes [23]. *In vitro *caMΦs have been shown to cause neuron death meanwhile aaMΦs appear to be less neurotoxic and possibly have some neurotrophic properties [24]. There is some evidence that T cells from MT-I/II^-/- ^mice are more responsive to stimuli that induce Th1 cells [25] and differences in the numbers of circulating leukocytes in MT-I/II^-/- ^mice relative to wild type mice have been observed [9]. Therefore, it is possible that the altered inflammatory response in the injured brain of MT-I/II^-/- ^mice is a result of an altered immune system but this has not been investigated in depth.

In the present study we used a cryolesion injury model to compare the number of T cells infiltrating the injury site in wild type and MT-I/II^-/- ^mice. Analysis of the numbers of leukocytes in circulation in the days following brain injury was also conducted to determine if the altered immune response to injury in MT-I/II^-/- ^mice occurs before the cells enter the injured brain. Levels of the mRNA marker of aaMΦs, Ym1, were assayed to determine if MT-I/II^-/- ^mice have different ratios of caMΦs/aaMΦs in comparison to wild type mice after brain injury.

## Methods

### Animals

All procedures involving animals were approved by the Animal Experimentation Ethics Committee of the University of Tasmania and were consistent with the Australian Code of Practice for the Care and Use of Animals for Scientific Purposes. Breeding stock for 129SI/SvImJ (wild type) mice and 129S7/SvEvBrd-*Mt1^tm1Bri ^Mt2^tm1Bri^*/J (MT-I/II^-/-^) mice [26] were obtained from Jackson Laboratories. Male mice from both strains were housed with food and water *ad libitum *with 12/12 hour light/dark cycling. Mice used in the experiment were between 12 and 36 weeks of age. For each experiment, mice from both strains were divided evenly into groups of 5-7 animals for sampling time (0, 1, 3 and 7 days post-injury, DPI) and animals within these groups were randomised and placed in numbered cages to blind the strain of the mouse from the researchers. Each mouse was housed in an individual cage for at least 3 days prior to injury surgery and during the period after surgery until euthanasia.

### Cryolesion brain injury

The cryolesion injury method was adapted from [27]. Mice were anaesthetised with 2-3% isoflurane/oxygen mix, delivered to the animal at 0.6 L/min. A 3 mm diameter, 50 mm long steel rod was cooled in liquid nitrogen. A sagittal incision along the skull was used to expose the cranium and the steel rod was then applied directly to the skull for 6 seconds. The stereotaxic coordinates for the injury site were 4 mm anterior of lambda and 2 mm right of the midline. The skin was sutured and the animal was allowed to recover back in its original cage. Mortality rate was less than 1% with a few animals showing signs of seizure within the first 24 hours after the application of the cryolesion injury. These animals were euthanized and excluded from the study.

### Immunohistochemistry

Mice were transcardially perfused with phosphate buffered saline (PBS); brains were dissected out of the skull and drop-fixed in 4% paraformaldehyde for 24 hours. The brains were embedded in wax and sectioned at 10 μm thickness. Before staining, antigen retrieval was undertaken in 0.01 M citrate buffer, pH 6, in a pressure cooker for 10 minutes. Primary antibodies used were 1:100 rat monoclonal NIMP-R14 to neutrophil (Abcam, Cambridge, UK) for neutrophils, 1:500 goat polyclonal anti-Iba1 (Abcam) for microglial/monocyte derived macrophages and 1:100 rabbit polyclonal anti-CD3 (Abcam) was used for T cells. All antibodies were diluted with 0.3% Triton-X 100 (Sigma, St. Lous, MO, USA) solution in PBS. Blocking with serum-free protein block (Dako, Glostrup, Denmark) was required for CD3 staining and was applied for 30 minutes before application of the primary antibody. The diluted NIMP-R14 antibody solution contained 10% normal goat serum (Vector Labs, Burlingame, CA, USA) as a blocking reagent. Biotinylated goat anti-rat IgG (Invitrogen, Carlsbad, CA, USA), biotinylated goat anti-rabbit IgG (Invitrogen) or donkey anti-goat IgG (Santa Cruz, Santa Cruz, CA USA) secondary antibodies, were applied to sections at 1:1000 dilution for 1 hour at room temperature. Avidin-biotin complex (Vector Labs) was used as the detection reagent and was applied to sections for 15 minutes followed by 2 rinses in PBS. Nickel enhanced 3'3-diaminobenzidine (DAB, Vector Labs) was used as the chromogen and was applied at the manufacturer's specified concentration for 8 minutes. Slides were then rinsed in distilled water for at least 5 minutes. Nuclear Fast Red (Sigma) was used as a counterstain for NIMP-R14 and Iba1 stained sections.

### Fluoro-jade C staining

Fluoro-jade C (Millipore, Billerica, MA, USA) is a neuron-specific marker of dead and degenerating neurons. Staining was carried out according to the protocol of Schmued et al. [28] whom demonstrated that fluoro-jade C labels both apoptotic and necrotic neuron death without discrimination. Rehydrated, slide-mounted sections were immersed in 0.06% potassium permanganate solution for 10 minutes. The slides were rinsed for 2 minutes in distilled water then immersed in 0.0001% fluoro-jade C, 0.01% acetic acid solution for 10 minutes. The slides were rinsed twice in distilled water for 5 minutes then were air-dried before they were coverslipped with DPX mounting medium (Merck, Whitehouse Station, NJ, USA).

### In situ cell counts in the injured brain

Low power, 10 × objective images were taken of the injury site for sections stained for fluoro-jade C, NIMP-R14, Iba1 and CD3. Fluoro-jade C counts were conducted for all positively labelled cells in the injury site and at lower depths in the un-injured CNS parenchyma. To standardise cell counts, fluoro-jade C counts were divided by the linear width of the injury in the section plane at the upper cortical surface in millimeters. NIMP-R14, Iba1 and CD3 positive cells were only counted within the injury site. Cell counts within the injury site were standardised to the area of the injury site in that section in mm^2^. The injury border was demarcated by an obvious degradation of tissue integrity observed in the injury site. The border of this region correlated well with the GFAP^+ ^endfeet extended by astrocytes as they re-established the glia limitans at days 3 and 7 DPI (data not shown). The glia limitans was not re-established at this injury border at 1 DPI but the pyknotic nuclei stained by nuclear fast red that likely represent apoptotic cells were rarely found outside this zone of reduced tissue integrity. Therefore this boundary was deemed to be a physiologically relevant demarcation of the injury zone. All cell counts were conducted blinded to the strain of the mouse.

### Flow cytometry

Blood was obtained by cardiac puncture and the leukocytes were separated from erythrocytes by Histopaque 1119 (Sigma) density gradient centrifugation. For CD3 and CD4 double labelling, 10^6 ^leukocytes were used for each batch of staining. Leukocytes were stained with a combination of 1 μg/ml APC-conjugated hamster IgG1 anti-mouse CD3e (BD Biosciences, Franklin Lakes, NJ, USA) and 1 μg/ml PE-conjugated rat IgG2a anti-mouse CD4 (BD biosciences) in 200 μl PBS-2%FCS at 4°C for 15 minutes. The cells were pelleted by centrifugation, and washed twice by resuspension in PBS-2%FCS followed by centrifugation to pellet. The pellet was resuspended in a fixation solution consisting of 2% paraformaldehyde, 4% D-glucose, 0.03% sodium azide and 0.01 M PBS for storage. For CD4^+^CD25^+^FoxP3^+ ^naturally occurring regulatory T cells, 10^6 ^cells were used for each batch of staining and were labelled with the mouse regulatory T cell staining kit # 2 (eBioscience, San Diego, CA, USA) according to the manufacturer's protocol. Staining procedures were also carried out for isotype control antibodies, PE-conjugated rat IgG2a (BD Biosciences) and APC-conjugated hamster IgG1 (BD Biosciences), which were applied at the same concentration as the specific antibodies to unstained cells. Samples were assayed by flow cytometry (BD Canto II flow cytometer) and were analysed using BD FACS Diva software v6.1.1. A quadratic gate was applied to the scatter plots of CD3 versus CD4 fluorescence to identify CD3^+^CD4^+ ^and CD3^+^CD4^- ^T cells which were expressed as a percentage of all leukocytes. To identify naturally occurring regulatory T cell populations, a gate was applied to cells expressing CD4. Cells within the CD4^+ ^population gate were analysed with a quadratic gate applied to the scatter plots of CD25 versus FoxP3. CD4^+^CD25^+^FoxP3^+ ^cells are expressed as a percentage of CD4^+ ^cells. The distinction between positive and negative staining was determined by the upper fluorescence of isotype control stained cells. All thresholds and gates were applied on this basis. To determine absolute circulating leukocyte numbers, blood was obtained from mice by cardiac puncture with syringes containing EDTA (3 mg per ml of blood). From each animal 250 μl whole blood was analysed in an Advia 120 haemocytological analyser (Siemens, Munich, Germany).

### Quantitative reverse-transcriptase PCR (RT-PCR)

Mice were transcardially perfused with PBS. The brain injury site was dissected out of the brain using a 3 mm biopsy punch and homogenised via Ultra-Turrax (IKA, Staufen, Germany) in TRI-reagent (Sigma, St. Louis, MO, USA). Peripheral blood mononuclear cells (PBMCs) were obtained using density gradient centrifugation on Histopaque 1083 (Sigma) and the pelleted cells from this fraction were homogenised in TRI-reagent. RNA was isolated from TRI-reagent according to the manufacturer's protocol. Reverse transcription with the Superscript-III reverse transcriptase system (Invitrogen) and quantitative PCR with Quantitect SYBR green (Qiagen, Hilden, Germany) was conducted according to the method of Brettingham-Moore et al. [29]. Oligonucleotide primers are detailed in table 1. The MT-I and MT-II primer sets were designed to be complementary to the cDNA for the transcripts from both wild type and MT-I/II^-/- ^mice, which still produce MT-I and MT-II transcripts but have premature stop codons inserted to prevent complete protein translation. Standard curves were created using known quantities of each PCR product and were used to determine the original cDNA copy number at an arbitrary fluorescence threshold (C_T_). GAPDH mRNA was used as the house keeping gene and MT-I and MT-II mRNA copy numbers were standardized to the copy number of the house-keeping gene, GAPDH.

**Table 1:** Oligonucleotide primer sets used for quantitative RT-PCR of brain mRNA samples after cryolesion brain injury.

Primer		Sequence (5' - 3')	**Accession No**.
GAPDH	Fwd	CCCAGAAGACTGTGGATGG	NM_008084.2
	Rev	GGATGCAGGGATGATGTTCT	

IFN-γ	Fwd	ACTGGCAAAAGGATGGTGAC	NM_008337.3
	Rev	GACCTGTGGGTTGTTGACCT	

IL-4	Fwd	TCAACCCCCAGCTAGTTGTC	NM_021283.2
	Rev	TCTGTGGTGTTCTTCGTTGC	

Ym1	Fwd	ACAATTTAGGAGGTGCCGTG	NM_009892.2
	Rev	CCAGCTGGTACAGCAGACAA	

MT-I	Fwd	GCTGTCCTCTAAGCGTCACC	NM_013602.3
	Rev	AGGAGCAGCAGCTCTTCTTG	

MT-II	Fwd	CAAACCGATCTCTCGTCGAT	NM_008630.2
	Rev	AGGAGCAGCAGCTTTTCTTG	

### Plasma cytokine assay

Blood was collected from mice via cardiac puncture with heparinised syringes. Plasma was obtained after centrifugation of blood for 5 minutes at 14000*g*. Plasma samples were diluted four-fold with PBS and assayed with a cytometric bead array mouse Th1/Th2/Th17 cytokine kit (BD biosciences). The assay was run according to specification and analysis was conducted using FCAP Array software v1.0.1. IL-4 and IFN-γ were assayed with ELISA kits (R&D systems, Minneapolis, MN, USA) according to manufacturer's protocol.

### Statistical Analysis

Statistical analysis was conducted with SPSS 15.0 (SPSS Inc.). Homogeneity of variances between groups within each data set was determined with Levene's test. Box-Cox test was used to determine the appropriate transformation for data sets with heterogeneous variances between groups. Statistical significance was determined with two-way ANOVA for p-values < 0.05 with Tukey's B Post-hoc test. All error bars in figures represent the standard error of the mean (SEM).

## Results

### Histological comparison of the extent of injury in wild type and MT-I/II^-/- ^mice

**Figure 1:**
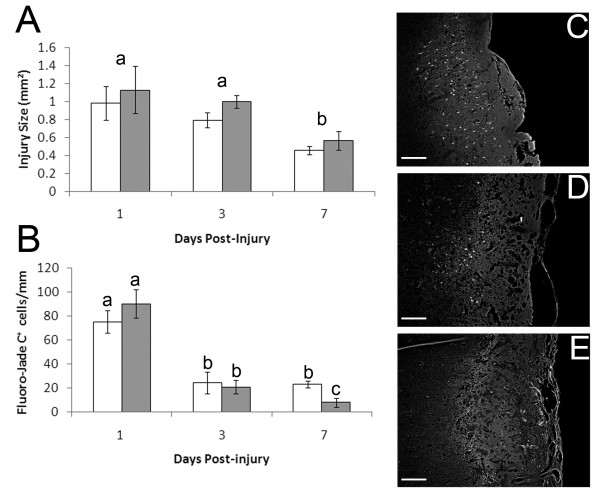
**Quantification of injury size and neuron death after cryolesion injury in wild type (grey bars) and MT-I/II^-/- ^mice (white bars)**. Injury size (A) was quantified by measurement of the area of the injury in sections taken from the widest point of the injury site. Neuron death identified by fluoro-jade C labelling (B). Fluorojade-C+ cells were counted in the injury site and the surrounding tissue. Counts were standardised per linear mm (width) of the injury site. Lower case letters indicate significance determined by Tukey's B post-hoc test. Time-points sharing letters indicates lack of statistically significant difference. n = 5-7, error bars = SEM. Representative images of fluoro-jade C staining in the injury site of wild type animals at 1 DPI (C), 3 DPI (D) and 7 DPI (E) with scale bars = 200 μm.

The area of the injury site in a 5 μm section taken from the widest point of the injury site was used as a comparative measure of injury size. The size of the injury did not change significantly from 1-3 DPI but declined significantly from 3-7 DPI in both wild type mice and MT-I/II^-/- ^mice (Figure 1A). No significant differences were observed between the strains at any time-point which suggests that on a large scale, the severity of the injury and rate of healing is similar in wild type and MT-I/II^-/- ^mice. However, investigation of the death of individual neurons revealed some notable differences between the two strains of mouse. Fluoro-jade C is a histological dye that labels neurons dying by both apoptosis and necrosis [28] and was found to label neurons in the lesion site and in the surrounding, uninjured parenchyma (Figure 1B). Quantification of fluoro-jade C labelled cells revealed that the highest degree of cell death occurred at 1 day post-injury (DPI, Figure 1B). In wild type mice the number of fluoro-jade C labelled cells decreased from 1-3 DPI and again at 3-7 DPI. MT-I/II^-/- ^mice had a similar decrease in fluoro-jade C staining from 1-3 DPI compared to wild type mice. In contrast to wild-type mice, the amount of cell death did not differ between 3 and 7 DPI in the injury site of MT-I/II^-/- ^mice. As a result, there were significantly greater numbers of fluoro-jade C labelled cells in MT-I/II^-/- ^mice at 7 DPI than in wild type mice.

### Leukocyte infiltration into the injury site in wild type and MT-I/II^-/- ^mice

**Figure 2:**
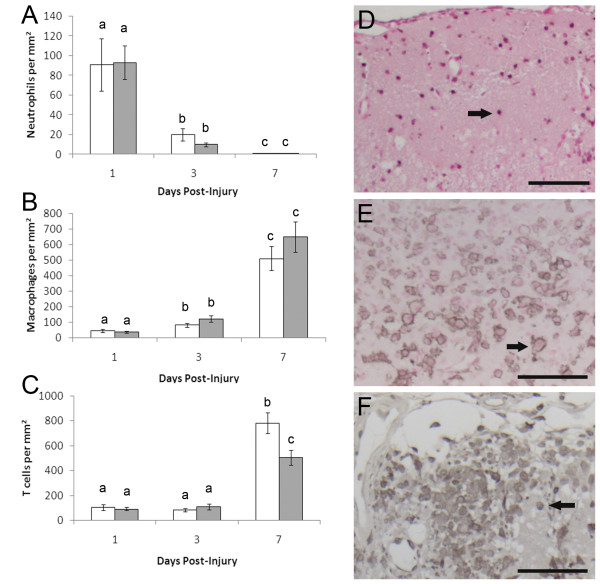
**Leukocyte counts in sections of the injury site of MT-I/II^-/- ^mice (white bars) and wild type mice (grey bars) standardised to injury area**. Neutrophil numbers (A) were determined by NIMP-14 immunoreactivity. Microglial and monocyte derived macrophages numbers (B) were determined by Iba1 immunoreactivity. T cell numbers (C) were determined by CD3 immunoreactivity. Lower case letters indicate significance determined by Tukey's B post-hoc test. Time-points sharing letters indicates lack of statistically significant difference. n = 5-7, error bars = SEM. Immunohistochemistry within the injury site is shown for neutrophils at 1 DPI with nuclear-fast red counter stain (D), macrophages at 7 DPI with nuclear-fast red counter stain (E) and T cells at 7 DPI without counterstain (D). Arrows indicate examples of positively stained cells, Scale bars = 100 μm.

Leukocyte infiltration into the cryolesion at 1, 3 and 7 DPI was investigated to determine if leukocytes could play a role in the sustained neuron death at 7 DPI in MT-I/II^-/- ^mice. Neutrophils were identified by immunohistochemistry for NIMP-14 and were at their highest levels in the injury site at 1 DPI coinciding with the highest amount of neuron death (Figure 2A).

Neutrophil numbers were greatly diminished at 3 DPI and mostly absent from the injury site at 7 DPI. No significant differences were found between neutrophil numbers in the injury site of wild-type and MT-I/II^-/- ^mice at 1, 3 or 7 DPI. Neutrophils were found mainly within the injury site but were occasionally found in the uninjured parenchyma proximal to the injury border.

Iba1 staining was used to identify macrophages derived from activated microglia and infiltrating monocytes within the injury site (Figure 2B). Macrophages within the injury site increased from 1-3 DPI and reached maximal numbers for the study period at 7 DPI. No significant differences in macrophage numbers between MT-I/II^-/- ^and wild type mice were observed at any time point.

CD3 antibody was used to identify T cells in the injury site (Figure 2C). T cells were found to be mainly confined to the injury site. T cell numbers were found to be relatively low at 1 and 3 DPI with no significant differences between injuries of wild type and MT-I/II^-/- ^mice. However, at 7 DPI, T cell numbers were greatly increased compared to the earlier time points. MT-I/II^-/- ^mice had significantly more T cells per mm^2 ^in the injury site than wild type mice at 7 DPI.

### Metallothionein expression in the cryolesion injury site

**Figure 3:**
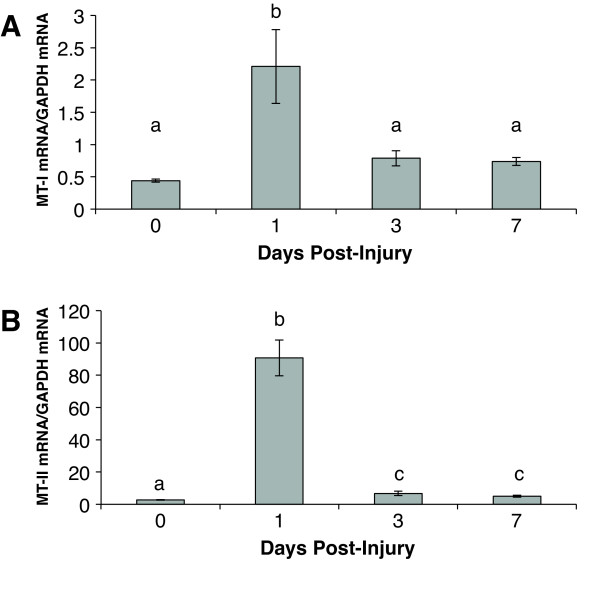
**MT-I and MT-II mRNA expression in wild type mice after cryolesion brain injury was measured by quantitative RT-PCR**. Peak expression for both mRNAs was observed at 1 DPI with statistical significance relative to uninjured animals. For all groups n = 7 except for wild type mice at zero DPI where n = 6, error bars = SEM.

The levels of MT-I and MT-II mRNA were assessed post-injury in wild type mice by quantitative RT-PCR. MT-I (Figure 3A) and MT-II (Figure 3B) mRNA both show significant increases in expression relative to GAPDH at 1 DPI. MT-II appears to be the dominant isoform of MT with 41.1 fold higher levels of expression than MT-I relative to GAPDH. At 3 and 7 DPI, MT-II mRNA was decreased but remained significantly elevated relative to the uninjured cortex, whereas MT-I mRNA had returned to pre-injury levels. It is interesting to note that peak MT-I and MT-II mRNA expression in the brain does not coincide with observed differences in neuron death and T cell infiltration in wild type and MT-I/II^-/- ^mice.

### Circulating leukocyte numbers in wild type and MT-I/II^-/- ^mice after brain injury

**Figure 4:**
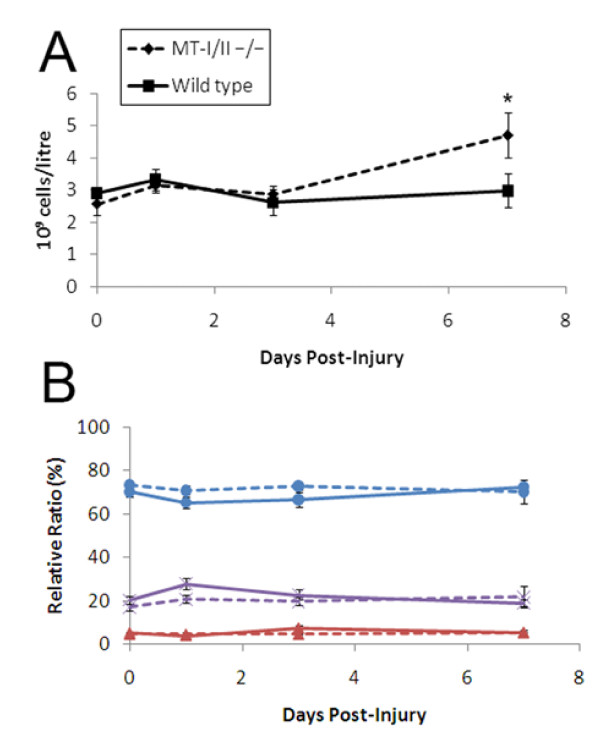
**Circulating leukocyte counts after brain injury were obtained with the Advia 120 haemocytological analyser**. Absolute cell numbers (A) show an increase at 7 DPI which was significantly different to all time points for wild type mice and from 0-3 DPI time points for MT-I/II^-/- ^mice as determined by Tukey's B post-hoc test. n = 4-6, error bars = SEM. Relative ratios of leukocytes (B) were compared between wild type mice (solid lines) and MT-I/II^-/- ^mice (dashed lines) for lymphocytes (blue circles), neutrophils (purple crosses) and monocytes (red triangles). No significant differences were found between strains for any cell type and no significant changes over time were found for any cell type. n = 4-6, error bars = SEM.

**Figure 5:**
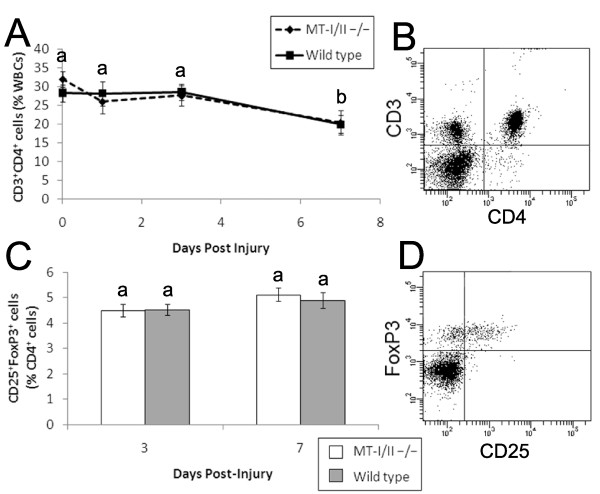
**CD4^+ ^T cell ratios after brain injury were assessed by flow cytometry for CD3 and CD4 labelled cells shown in a representative scatter plot (B)**. Temporal changes in CD4^+ ^T cell ratios after brain injury reveal no significant differences between wild type (solid line) and MT-I/II^-/- ^mice (dashed line) (A), n = 6-7, error bars = SEM. The CD4^+ ^cell gate revealed the ratios of CD25^+ ^and FoxP3^+ ^naturally occurring regulatory T cells (C). At 3 and 7 DPI no significant differences were observed between wild type mice (grey bars) and MT-I/II^-/- ^mice (white bars) (D), n = 7, error bars = SEM.

Previous studies have found MT-I/II^-/- ^mice to have altered levels of circulating leukocytes and leukocyte sub-types [9]. A haematological analyser was used to determine whether differences in absolute numbers of white blood cells in MT-I/II^-/- ^mice and wild type mice might account for differences in leukocyte infiltration rates into the cryolesion-affected tissue (Figure 4A). Leukocyte counts from whole peripheral blood did not significantly change after brain injury in wild type mice at 0 (uninjured), 1, 3 or 7 DPI. MT-I/II^-/- ^mice had no significant changes in leukocyte numbers in whole peripheral blood from 0-3 DPI but had significantly higher leukocyte counts at 7 DPI compared to uninjured controls and wild type mice at 7 DPI (Figure 4A). Analysis of circulating neutrophils, lymphocytes and monocytes revealed no differences in the relative ratios of any leukocyte sub-type (Figure 4B). Basophils and eosinophils constituted a small fraction of all leukocytes and numbers did not increase after injury in MT-I/II^-/- ^mice and do not explain the increased levels of leukocytes in MT-I/II^-/- ^mice at 7 DPI (data not shown). Because the haematological analyser did not differentiate between sub-classes of lymphocytes, flow cytometry was used to determine if relative ratios of T cells were equal in MT-I/II^-/- ^mice and wild type mice (Figure 5A). By 7 DPI, there was a significant overall decrease in CD3^+^CD4^+ ^helper T cells in both MT-I/II^-/- ^mice and wild type mice. However, there was no significant difference at any timepoint between these two groups of mice. There was also no difference in numbers of CD3^+^CD4^- ^T cells, the majority of which are likely to consist of cytotoxic T cells (data not shown).

CD4^+^CD25^+^FoxP3^+ ^naturally occurring regulatory T cells have been shown to reduce the impact of stroke [30] and may have similar protective roles in the injured brain. However, the number of CD4^+^CD25^+^FoxP3^+ ^naturally occurring regulatory T cells as a percentage of CD4^+ ^T cells was not found to vary significantly at 3 or 7 DPI between wild type and MT-I/II^-/- ^mice (Figure 5C). Therefore, there were no differences in the ratios of any of the leukocyte sub-types investigated in wild type and MT-I/II^-/- ^mice after brain injury. However, the increase in absolute leukocyte numbers observed in MT-I/II^-/- ^mice at 7 DPI allows us to calculate that there would be an average increase of 16% in the absolute number of circulating T cells in MT-I/II^-/- ^mice at 7 DPI. Because there was no increase in the absolute number of leukocytes in wild type mice, the same calculation determines that absolute numbers of circulating T cells would have decreased by 26% on average by 7 DPI. This result is in accordance with the finding that T cell numbers are lower in the brain of wild type mice compared to MT-I/II^-/- ^mice at 7 DPI.

### Comparison of chemokine and cytokine expression in wild type and MT-I/II mice

**Figure 6:**
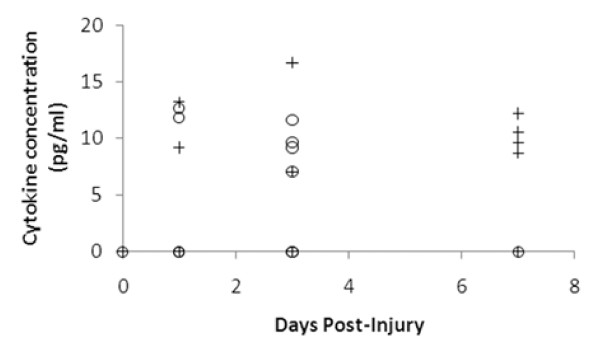
**Scatter plot showing detectable plasma IL-2 concentrations in MT-I/II^-/- ^mice (crosses) and wild type mice (circles) after brain injury**. Values below the detection limit (0.4 pg/ml) are not shown. Increases in IL-2 in plasma after injury were sporadic with few animals posting detectable concentrations. At 7 DPI only MT-I/II^-/- ^mice have detectable levels of plasma IL-2. n = 7 for all groups except wild type mice at zero DPI and MT-I/II^-/- ^mice at 7 DPI for which n = 6, error bars = SEM.

Plasma concentrations of Th1 and Th2 cytokines were assayed to determine if there were systemic differences in the inflammatory response to brain injury. Unfortunately most of the cytokines tested were not detectable or were only rarely detected (IL-4, IL-6, INF-γ, TNF-α and IL-10) which suggests that the cryolesion does not induce a strong systemic inflammatory response. However, it is known that T cells must be activated before they can enter the CNS [31,27] and the cytokine, IL-2 which is responsible for T cell activation, was detectable in the plasma of some animals after injury (Figure 6). At 1 and 3 DPI, IL-2 was detected in some animals from both the wild type and MT-I/II^-/- ^groups. At 7 DPI, only MT-I/II^-/- ^mice had detectable plasma concentrations of IL-2 with 4 out of 6 animals exhibiting detectable expression of IL-2. No wild type animals had detectable levels of IL-2 at 7 DPI. Statistical analysis could not be conducted on this data set due to the high number of animals with plasma IL-2 concentrations lower than the detection limit.

IL-4 and IFN-γ mRNA could not be detected in the injury site of either strain of mouse, yet both transcripts were detectable in RNA harvested from a mouse T cell line that had been stimulated with calcium ionophore and phorbol ester to induce a state of activation (data not shown). IL-4 and IFN-γ protein could not be detected in the injury site of MT-I/II^-/- ^mice and wild type mice. Overall, systemic cytokine activation was not observed after brain injury and local T cell specific cytokines were undetectable so Th1 and Th2 responses could not be compared directly in wild type and MT-I/II^-/- ^mouse injury sites.

**Figure 7:**
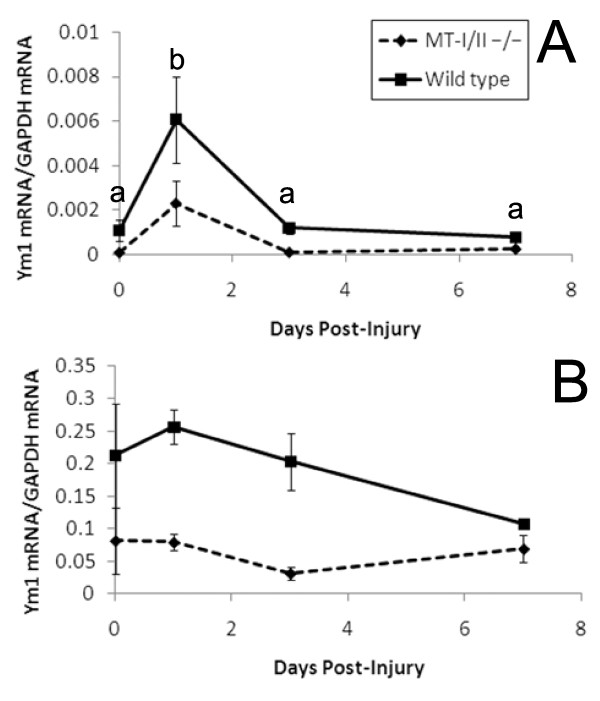
**(A) Ym1 mRNA expression is greater in the injury site of wild type mice (solid lines) than in MT-I/II^-/- ^mice (dashed lines), n = 6-7, error bars = SEM**. (B) Ym1 mRNA expression is significantly greater in the circulating PBMCs of wild type mice (Solid lines) than in MT-I/II^-/- ^mice (dashed lines), independent of time after injury, n = 6, error bars = SEM.

To assess the effect of increased numbers of T cells in the injury site, quantitative RT-PCR was used to assess the levels of mRNA for the alternative macrophage activation marker, Ym1, in the cryolesion site (Figure 7A). Ym1 expression increased significantly at 1 DPI in the injury site of wild type and MT-I/II^-/- ^mice as determined by 2-way ANOVA. It was also determined that wild type mice have significantly higher levels of Ym1 compared to MT-I/II^-/- ^mice independent of the factor of time before or after injury.

Using this method, it was impossible to determine whether the Ym1 is derived from the CNS-resident microglia or infiltrating monocytes because both cell types contribute to the pool of activated macrophages in the injury site. To examine whether the increased Ym1 in wild type mice occurs in monocytes before they enter the injured brain, RT-PCR was used to determine Ym1 levels in PBMCs (Figure 7B). Monocytes are the only cell type in this cell fraction that express Ym1. 2-way ANOVA revealed that there were no significant changes in Ym1 expression over time, but wild type animals express significantly higher levels of Ym1 mRNA in their PBMCs compared to MT-I/II^-/- ^mice.

## Discussion

The present study demonstrates that the altered immune response present in MT-I/II^-/- ^mice occurs in later stages of brain injury. The significant findings of increased T cell infiltrate into the injury site, increased levels of IL-2, prolonged neuronal death surrounding the injury site and increased numbers of circulating leukocytes were all only present in MT-I/II^-/- ^mice at 7 DPI. However, the finding that MT-I/II^-/- ^mice have altered expression of Ym1 mRNA in the brain and circulating monocytes, both before and after injury, suggests that at least some aspects of the altered immune response in MT-I/II^-/- ^mice are independent of brain injury. The implication of these findings is that MT-I/II may be having effects on the immune system systemically hence MT-I/II may be affecting the progression of brain injury indirectly via modulation of inflammatory responses.

A latent period before increased neuron death in MT-I/II^-/- ^mice compared to wild type mice has been reported previously [1] which is in accordance with the findings of the present study. However, increased neuron death in MT-I/II^-/- ^mice has also been observed within 24 hours of brain injury by Penkowa et al. [2]. One of the criticisms of the study by Penkowa et al. [2] is that neuron death in the cryolesioned cortex was assessed by counting the number of remaining neuron-specific enolase labelled neurons immediately adjacent to the injury border. However in that study, the size of injury site was larger in MT-I/II^-/- ^mice compared to the wild type controls, hence neuron counts from MT-I/II^-/- ^mice would have come from deeper cortical layers than in wild type mice. It is well known that there are large differences in the density and distribution of neurons in different layers of the cortex [32]. Natale et al. [1] also used counts of remaining neurons to measure neuron death in brain injury but their method of brain injury allowed counting to be conducted in the same region in both wild type MT-I/II^-/- ^mice. In the present study there were no significant differences between injury sizes in wild type and MT-I/II^-/- ^mice and cell counts were conducted based on a specific marker of neuronal death.

Crowthers et al. [9] have reported that MT-I/II^-/- ^mice have altered numbers of T cells in the blood and spleen when compared to wild type mice but other investigators found no difference in T cell numbers in the spleen and lymph nodes of wild type and MT-I/II^-/- ^mice [25]. In the present study, leukocyte numbers in MT-I/II^-/- ^mice were only found to differ after brain injury. Extracellular MT-I/II has been shown to have modulatory effects on multiple types of leukocytes [9-14,33,34]. Increases in extracellular MT have been observed in the blood of head injured patients despite the fact that MT-I/II protein has no secretory signal sequence [35]. However, the concentration of MT-I/II required to modulate the activity of immune cells *in vitro *is often higher than the levels of MT observed in circulation before or after head injury so further study is required to determine whether this mechanism is possible under the physiological conditions that occur after brain injury.

A possible mechanism by which MT-I/II acts on the immune system that has received little attention is that the zinc-binding ability of MT-I/II may affect immune system functioning. Zinc supplementation in humans has been shown to enhance leukocyte responses to activating stimuli [36] and zinc homeostasis is known to be disrupted by brain injury [37]. We have recently demonstrated that zinc is released from hepatic stores in mice after brain injury and that MT-I/II^-/- ^mice have a reduced capacity for zinc sequestration to the liver, a process that occurs at 7 DPI in wild type mice (Pankhurst et al., manuscript submitted). The co-occurrence of this event with many of the altered immune system responses observed at 7 DPI in the present study provides evidence that MT-I/II mediated zinc homeostasis may be linked to immune system functioning.

We can not exclude the possibility that MT-I/II is interacting with more than one process that affects the injured brain. Increased Ym1 mRNA expression is a marker for aaMΦs [38] and was found to be significantly higher in wild type mice than MT-I/II^-/- ^mice in both the injury site and PBMCs. The fact that Ym1 was higher in wild type mice compared to MT-I/II^-/- ^mice before injury implies that wild type macrophages have a greater intrinsic disposition to become aaMΦs than those from MT-I/II^-/- ^mice. This intriguing observation is likely to be independent of effects on zinc homeostasis in the liver of mice lacking MT-I/II. This trend was retained throughout the period after brain injury and may be partly responsible for the increased neuron death that was observed at 7 DPI in MT-I/II^-/- ^mice compared to wild type mice. *In vitro*, the aaMΦ response has been shown to be much less neurotoxic than the caMΦ response which is purported to be due to the higher production of reactive oxygen species by caMΦs [24]. CaMΦs also produce higher levels of neurotoxic metabolites via the quinolinic acid pathway [39,40]. Th1 cytokines are responsible for the generation of caMΦs and Th2 cytokines are responsible for the generation of aaMΦs. We have previously shown that exogenous application of MT-I/II to the injured rat brain leads to a reduction in quinolinic acid production and extracellular application of MT-I/II to cultured microglia reduces Th1 cytokine-mediated (IFN-γ) production of quinolinic acid [41]. This is supported by the finding that naive T cells isolated from MT-I/II^-/- ^mice have been shown to be more responsive to becoming Th1 cells than T cells from wild type mice [25]. One limitation of the present study is that measurement of the Th1/Th2 responses were not possible and we have only provided a single marker of aaMΦs hence more experiments are required to determine if differential macrophage activation is occurring in MT-I/II^-/- ^mice and whether Th1/Th2 ratios differ. Immunoassay and RT-PCR of cytokines were not sensitive enough to definitively determine the relative ratios of the Th1 and Th2 responses in the injured brain in the present study. This was most likely due to the small tissue sample sizes and the fact that cytokines can operate at very low concentrations. However, IL-2 was detectable in the plasma of some animals and it was interesting to find that at 7 DPI only MT-I/II^-/- ^mice were producing detectable amounts of IL-2. IL-2 is responsible for the clonal expansion of activated T cells [42] and we regard this as evidence that T cell activity was altered in MT-I/II^-/- ^mice after brain injury and may explain the differences observed in T cell infiltration in MT-I/II^-/- ^mice.

## Conclusions

MT-I/II expression is increased in the brain after brain injury which suggests that some of the protective effects of MT-I/II after brain injury are acting directly on the injured brain. However, many of the processes observed in the current study are initiated outside the CNS. Therefore, it is possible that MT-I/II produced outside the injured brain could be more important for the modulation of immune response after brain injury than MT-I/II produced within the CNS. Such an interaction would increase the prospects for the use of MT-I/II as a therapeutic for brain injury.

## Competing interests

The authors declare that they have no competing interests.

## Authors' contributions

MWP conceived the experimental design and conducted the majority of experimental procedures and statistical analysis. WB developed the immunohistochemical techniques for the study and participated in experimental procedures. AKW, MTKK and RSC were involved in the development of the experimental approach and contributed significantly to interpretation of data and preparation of the manuscript. All authors have read and approved the final manuscript.

## References

[B1] Natale JE Knight JB Cheng Y Rome JE Gallo V Metallothionein I and II mitigate age-dependent secondary brain injury J Neurosci Res 2004 78 303 314 10.1002/jnr.20265 15389833

[B2] Penkowa M Carrasco J Giralt M Moos T Hidalgo J CNS wound healing is severely depressed in metallothionein I and II-deficient mice J Neurosci 1999 19 2535 2545 10087067 10.1523/JNEUROSCI.19-07-02535.1999 PMC6786080

[B3] Potter EG Cheng Y Natale JE Deleterious Effects of Minocycline After In Vivo Target Deprivation of Thalamocortical Neurons in the Immature, Metallothionein-deficient Mouse Brain J Neurosci Res 2009 87 1356 1368 10.1002/jnr.21963 19115404 PMC4333151

[B4] Dineley KE Scanlon JM Kress GJ Stout AK Reynolds IJ Astrocytes are more resistant than neurons to the cytotoxic effects of increased [Zn2+](i) Neurobiol Dis 2000 7 310 320 10.1006/nbdi.2000.0303 10964603

[B5] Suzuki Y Apostolova MD Cherian MG Astrocyte cultures from transgenic mice to study the role of metallothionein in cytotoxicity of tert-butyl hydroperoxide Toxicology 2000 145 51 62 10.1016/S0300-483X(99)00220-6 10771131

[B6] Chung RS Vickers JC Chuah MI West AK Metallothionein-IIA promotes initial neurite elongation and postinjury reactive neurite growth and facilitates healing after focal cortical brain injury J Neurosci 2003 23 3336 3342 12716941 10.1523/JNEUROSCI.23-08-03336.2003 PMC6742325

[B7] Penkowa M Giralt M Moos T Thomsen PS Hernandez J Hidalgo J Impaired inflammatory response to glial cell death in genetically metallothionein-I- and -II-deficient mice Exp Neurol 1999 156 149 164 10.1006/exnr.1998.7009 10192786

[B8] Potter EG Cheng Y Knight JB Gordish-Dressman H Natale JE Metallothionein I and II attenuate the thalamic microglial response following traumatic axotomy in the immature brain J Neurotrauma 2007 24 28 42 10.1089/neu.2006.0056.R1 17263668

[B9] Crowthers KC Kline V Giardina C Lynes MA Augmented humoral immune function in metallothionein-null mice Toxicol Appl Pharmacol 2000 166 161 172 10.1006/taap.2000.8961 10906280

[B10] Canpolat E Lynes MA In vivo manipulation of endogenous metallothionein with a monoclonal antibody enhances a T-dependent humoral immune response Toxicol Sci 2001 62 61 70 11399794 10.1093/toxsci/62.1.61

[B11] Lynes MA Borghesi LA Youn JH Olson EA Immunomodulatory activities of extracellular metallothionein. 1. Metallothionein effects on antibody-production Toxicology 1993 85 161 177 10.1016/0300-483X(93)90040-Y 8303711

[B12] Mita M Imura N Kumazawa Y Himeno S Suppressed proliferative response of spleen T cells from metallothionein null mice Microbiol Immunol 2002 46 101 107 11939574 10.1111/j.1348-0421.2002.tb02665.x

[B13] Borghesi LA Youn J Olson EA Lynes MA Interactions of metallothionein with murine lymphocytes: Plasma membrane binding and proliferation Toxicology 1996 108 129 140 10.1016/S0300-483X(95)03243-9 8644111

[B14] Emeny RT Marusov G Lawrence DA Pederson-Lane J Yin X Lynes MA Manipulations of metallothionein gene dose accelerate the response to Listeria monocytogenes Chem Biol Interact 2009 181 243 253 10.1016/j.cbi.2009.06.018 19576872 PMC12990889

[B15] Stirling DP Yong VW Dynamics of the inflammatory response after murine spinal cord injury revealed by flow cytometry J Neurosci Res 2008 86 1944 1958 10.1002/jnr.21659 18438914

[B16] Czigner A Mihaly A Farkas O Buki A Krisztin-Peva B Dobo E Barzo P Kinetics of the cellular immune response following closed head injury Acta Neurochirurgica 2007 149 281 289 10.1007/s00701-006-1095-8 17288002

[B17] Clausen F Lorant T Lewen A Hillered L T lymphocyte trafficking: A novel target for neuroprotection in traumatic brain injury J Neurotrauma 2007 24 1295 1307 10.1089/neu.2006.0258 17711391

[B18] Sroga JM Jones TB Kigerl KA McGaughy VM Popovich PG Rats and mice exhibit distinct inflammatory reactions after spinal cord injury J Comp Neurol 2003 462 223 240 10.1002/cne.10736 12794745

[B19] Bezzi P Domercq M Brambilla L Galli R Schols D De Clercq E Vescovi A Bagetta G Kollias G Meldolesi J Volterra A CXCR4-activated astrocyte glutamate release via TNFa: amplification by microglia triggers neurotoxicity Nat Neurosci 2001 4 702 710 10.1038/89490 11426226

[B20] Desagher S Glowinski J Premont J Astrocytes protect neurons from hydrogen peroxide toxicity J Neurosci 1996 16 2553 2562 8786431 10.1523/JNEUROSCI.16-08-02553.1996 PMC6578753

[B21] Hu JR Ferreira A VanEldik LJ S100 beta induces neuronal cell death through nitric oxide release from astrocytes J Neurochem 1997 69 2294 2301 9375660 10.1046/j.1471-4159.1997.69062294.x

[B22] Mosmann TR Cherwinski H Bond MW Giedlin MA Coffman RL 2 types of murine helper T-cell clone. 1. Definition according to profiles of lymphokine activities and secreted proteins J Immunol 1986 136 2348 2357 2419430

[B23] Mills CD Kincaid K Alt JM Heilman MJ Hill AM M-1/M-2 macrophages and the Th1/Th2 paradigm J Immunol 2000 164 6166 6173 28923981 10.4049/jimmunol.1701141

[B24] Kigerl KA Gensel JC Ankeny DP Alexander JK Donnelly DJ Popovich PG Identification of Two Distinct Macrophage Subsets with Divergent Effects Causing either Neurotoxicity or Regeneration in the Injured Mouse Spinal Cord J Neurosci 2009 29 13435 13444 10.1523/JNEUROSCI.3257-09.2009 19864556 PMC2788152

[B25] Huh S Lee K Yun HS Paik DJ Kim JM Youn J Functions of metallothionein generating interleukin-10-producing regulatory CD4(+) T cells potentiate suppression of collagen-induced arthritis J Microbiol Biotech 2007 17 348 358 18051768

[B26] Masters BA Kelly EJ Quaife CJ Brinster RL Palmiter RD Targeted disruption of metallothionein-i and metallothionein-II genes increases sensitivity to cadmium Proc Natl Acad Sci USA 1994 91 584 588 10.1073/pnas.91.2.584 8290567 PMC42993

[B27] Ling CY Sandor M Suresh M Fabry Z Traumatic injury and the presence of antigen differentially contribute to T-cell recruitment in the CNS J Neurosci 2006 26 731 741 10.1523/JNEUROSCI.3502-05.2006 16421293 PMC6675378

[B28] Schmued LC Stowers CC Scallet AC Xu LL Fluoro-Jade C results in ultra high resolution and contrast labeling of degenerating neurons Brain Res 2005 1035 24 31 10.1016/j.brainres.2004.11.054 15713273

[B29] Brettingham-Moore KH Rao S Juelich T Shannon MF Holloway AF GM-CSF promoter chromatin remodelling and gene transcription display distinct signal and transcription factor requirements Nucleic Acids Research 2005 33 225 234 10.1093/nar/gki161 15647505 PMC546149

[B30] Liesz A Suri-Payer E Veltkamp C Doerr H Sommer C Rivest S Giese T Veltkamp R Regulatory T cells are key cerebroprotective immunomodulators in acute experimental stroke Nat Med 2009 15 192 199 10.1038/nm.1927 19169263

[B31] Byram SC Carson MJ DeBoy CA Serpe CJ Sanders VM Jones KJ CD4-positive T cell-mediated neuroprotection requires dual compartment antigen presentation J Neurosci 2004 24 4333 4339 10.1523/JNEUROSCI.5276-03.2004 15128847 PMC2665301

[B32] DeFelipe J Alonso-Nanclares L Arellano JI Microstructure of the neocortex: Comparative aspects J Neurocytol 2002 31 299 316 10.1023/A:1024130211265 12815249

[B33] Youn J Lynes MA Metallothionein-induced suppression of cytotoxic T lymphocyte function: an important immunoregulatory control Toxicol Sci 1999 52 199 208 10.1093/toxsci/52.2.199 10630572

[B34] Youn J Borghesi LA Olson EA Lynes MA Immunomodulatory activities of extracellular metallothionein. II. Effects on macrophage functions J Toxicol Env Health 1995 45 397 413 10.1080/15287399509532004 7643428

[B35] Kukačka J Vajtr D Huska D Prusa R Houstava L Samal F Diopan V Kotaska K Kizek R Blood metallothionein, neuron specific enolase, and protein S100B in patients with traumatic brain injury Neuroendocrinol Lett 2006 27 116 120 17159794

[B36] Aydemir TB Blanchard RK Cousins RJ Zinc supplementation of young men alters metallothionein, zinc transporter, and cytokine gene expression in leukocyte populations Proc Natl Acad Sci USA 2006 103 1699 1704 10.1073/pnas.0510407103 16434472 PMC1413653

[B37] McClain CJ Twyman DL Ott LG Rapp RP Tibbs PA Norton JA Kasarskis EJ Dempsey RJ Young B Serum and urine zinc response in head-injured patients J Neurosurg 1986 64 224 230 10.3171/jns.1986.64.2.0224 3944632

[B38] Raes G De Baetselier P Noel W Beschin A Brombacher F Hassanzadeh G Differential expression of FIZZ1 and Ym1 in alternatively versus classically activated macrophages J Leukocyte Biol 2002 71 597 602 11927645

[B39] Kwidzinski E Bechmann I IDO expression in the brain: a double-edged sword J Mol Med 2007 85 1351 1359 10.1007/s00109-007-0229-7 17594069

[B40] Yadav MC Burudi EME Alirezaei M Flynn CC Lanigan CM Fox HS IFN-gamma-induced IDO and WRS expression in microglia is differentially regulated by IL-4 Glia 2007 55 1385 1396 10.1002/glia.20544 17661345 PMC2486430

[B41] Chung RS Leung YK Butler CW Chen Y Eaton ED Pankhurst MW West AK Guillemin GJ Metallothionein Treatment Attenuates Microglial Activation and Expression of Neurotoxic Quinolinic Acid Following Traumatic Brain Injury Neurotox Res 2009 15 381 389 10.1007/s12640-009-9044-y 19384571

[B42] Malek TR The Biology of Interleukin-2 Annu Rev Immunol 2008 26 453 79 10.1146/annurev.immunol.26.021607.090357 18062768

